# Acute subdural hematoma post ventricular puncture in infants: A case report and review of the literature

**DOI:** 10.1016/j.ijscr.2021.105913

**Published:** 2021-04-27

**Authors:** El Hadji Cheikh Ndiaye Sy, Yakhya Cisse, Jean Michel Nzisabira, Ansoumane Donzo, Pape Sandene Ndiaye, Seydou Boubakar Badiane

**Affiliations:** Neurosurgery Department, Fann University Hospital Center, Dakar, Senegal

**Keywords:** Acute subdural hematoma, Ventricular puncture, Infant, Decompressive craniectomy

## Abstract

**Introduction and importance:**

Acute subdural hematoma in infants is often due to non-accidental causes such as shaken baby syndrome or abuse. Occasionally a rupture of the cerebral bridge veins after ventricular puncture can lead to a subdural hematoma in infant. In this article we report the very first case of acute subdural hematoma after ventricular puncture of cerebrospinal fluid.

**Case presentation:**

It is a 40-day-old male infant received at the pediatric emergency room for an infectious syndrome. An etiological assessment was carried out including a ventricular puncture of the cerebrospinal fluid. Two days after the puncture, the child develops a sudden alteration of consciousness during hospitalization, with a Blantyre coma score of 3/5. The CT scan performed showed a right subdural parieto-temporal hematoma associated with a right fronto-temporal parietal parenchymal hypodensity. A right temporo-parietal decompressive craniectomy was performed with evacuation of the acute subdural hematoma. Clinical improvement was obtained and the child was discharged after 3 weeks of hospitalization.

**Clinical discussion:**

Acute subdural hematoma post ventricular puncture is rarely reported in the literature. The mechanism would probably be a rupture of the bridging veins by sudden collapse of the parenchyma following rapid and excessive aspiration of cerebrospinal fluid. Its management is medico-surgical. This manuscript further demonstrates the importance of mastering the ventricular puncture technique which must be performed by an experienced neurosurgeon.

**Conclusion:**

The ventricular puncture remains a delicate gesture which must be carried out by a qualified neurosurgeon because of the risks of complications such as an acute subdural hematoma.

## Highlights

• Infant ventricular puncture is a diagnostic and therapeutic procedure that must be performed by an experienced neurosurgeon; aspiration of cerebrospinal fluid should be done slowly.
• Rarely this puncture is complicated by an acute subdural hematoma as in our case.
• The mechanism of occurrence of an acute subdural hematoma in infants is different from the mechanisms in older children and adults
• The management of a subdural hematoma post ventricular puncture is interdisciplinary.
• Our treatment consisted of performing a decompression flap and evacuation of the acute subdural hematoma.

## 1 Introduction

Subdural hematoma in infants is distinct from that occurring in children or adults due in particular to their mechanism of occurrence [1]. The aetiologies are mainly traumatic and among these are non-accidental causes such as shaken baby syndrome or abuse. A rupture of the cerebral bridge veins is also described [2]. In this article, we report the very first case of an acute subdural hematoma post ventricular cerebrospinal fluid puncture (CSF), in a 40-day-old infant, treated at the Fann University Hospital in Dakar. We will discuss the mechanism of hematoma formation and treatment. This case report has been reported in line with the SCARE 2020 Criteria [3].

## 2 Case presentation

**Fig. 1:**
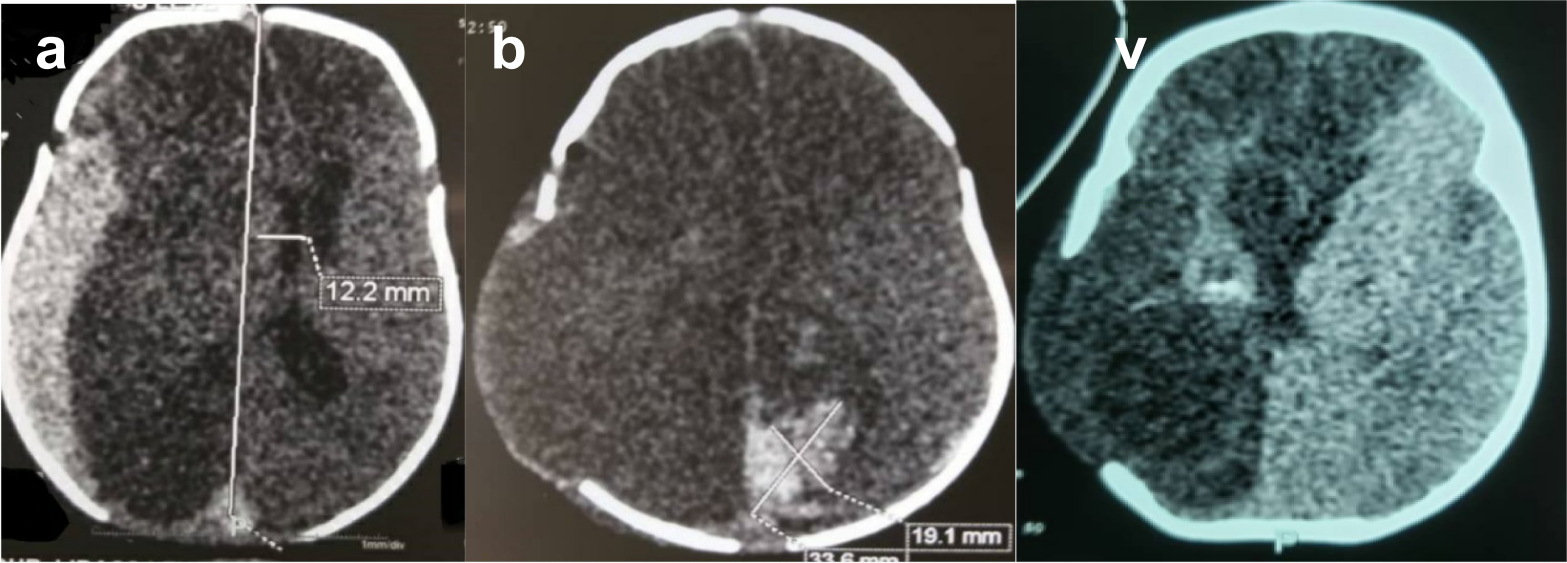
Brain CT scan of a 40-day-old infant showing an acute right parietotemporal subdural hematoma with mass effect (1a). post-decompressive brain CT scan, note the occipital intra-parenchymal hematoma (1b). Control brain CT scan taken 4 months earlier showing sequelae ischemia and the area of craniectomy (1c).

**Fig. 2:**
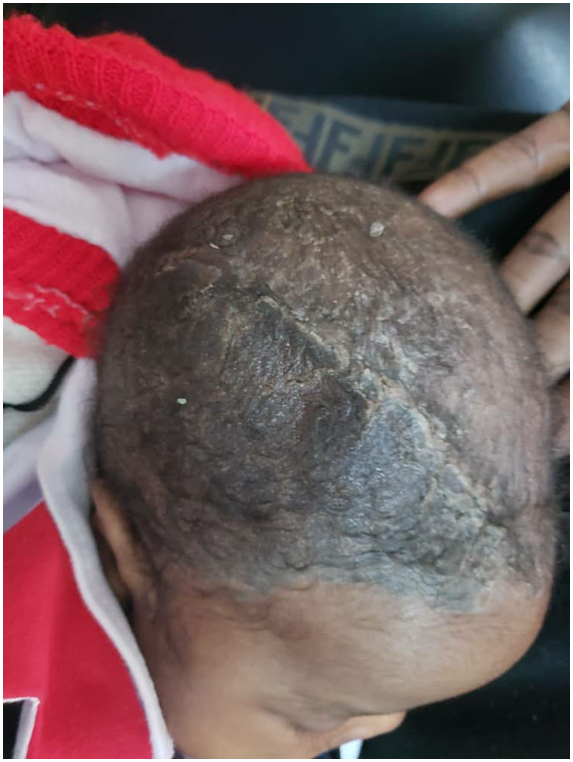
Zone of decompressive craniectomy.

This is a 40-day-old male infant with up-to-date vaccination status, no medical and family history of illness, or surgery; received at Fann's pediatric emergency room, for a febrile convulsion. The initial general examination found a temperature of 38.2 °C, a weight of 4500 g, a height of 60 cm, a BMI at 25.51Kg/m2, a head circumference at 42 cm, a heart rate at 150 Bpm and a frequency respiratory at 37 cpm. The remainder of the examination found an anterior fontanel that was slightly strained and a neck that is soft on examination. The full blood count performed initially was normal, the C-Reactive protein was 32.20 mg/l. The procalcitonin assay showed a slightly elevated level at 2.41 mg/ml (normal less than 0.5 mg/ml). The chest x-ray was normal. A complement of the etiological assessment was carried out including a ventricular puncture of the CSF with cytochemical and bacteriological analysis. Then was put on medical treatment. Two days after the puncture, the child had developed a sudden alteration of consciousness during hospitalization. Neurological examination revealed a Blantyre coma score of 3/5, pupils of normal sizes not very reactive to lights stimulation, he had a mobilization of the 4 limbs to the stimulation which was easier on the right, the reflexes were present and normal. The rest of the exam was normal. The brain scan performed showed a right subdural parieto-temporal hematoma associated with a right fronto-temporo-parietal parenchymal hypodensity with mass effect on neighboring cerebral structures (Fig. 1a). The infant was immediately taken to intensive care unit. An indication of evacuation of the hematoma was asked and performed on the same day after obtaining a signed informed consent of his parents. The time between the last clinical presentation and the surgical intervention was 3 h. The infant, under general anesthesia and nasotracheal intubation, was covered and placed in the supine position. He was operated on by an assistant clinical chief neurosurgeon. He underwent a decompressive craniectomy with evacuation of the hematoma and duroplasty without conservation of the flap. Intraoperatively, he received a blood transfusion. The postoperative period was marked by a slight delay in waking up. On examination, the child was hemodynamically and respiratory stable, the Blantyre score was 4/5, the pupils was intermediate and reactive. The early control CT scan showed sequelae right hemispherical ischemia, a cerebral herniation associated with an intraparenchymal hematoma (Fig. 1b). Clinical improvement was obtained later and the infant was discharged after 3 weeks of hospitalization with regular follow-up. At 4 months of follow-up, the neurological examination found a good Blantyre score of 5/5, the pupils were reactive, he spontaneously mobilized his 4 limbs. Examination of the cephalic extremity found a scar on the area of decompressive craniectomy (Fig. 1c). The control cerebral CT scan showed right hemispherical sequelae ischemia without mass effect, a cerebral herniation and a ventricular dilation (Fig. 2). After 4 months of follow-up, the parents expressed their satisfaction with the care of their child.

## 3 Discussion

Acute subdural hematoma in infants is frequently of traumatic origin. Non-accidental causes include shaken baby syndrome or child abuse. This results in severe brain lesions in the infant, of which the subdural hematomas occupy a considerable part [4]. Occasionally, the subdural hematoma may result from a rupture of the bridge veins [5]. In our case, the latter etiology was retained as the mechanism for the occurrence of the hematoma. Indeed, the infant underwent a ventricular puncture of CSF for laboratory analyses. Our hypothesis is a significant withdrawal of CSF having led to a collapse of the cerebral parenchyma causing the rupture of the veins bridge to dural destination thus forming the hematoma.

In infants, the subdural hematoma is clinically characterized by convulsive seizures, disorders of consciousness, retinal hemorrhage or apnea in moderate forms which can go as far as coma with hemiplegia or even death. [6]. If intracranial hypertension is associated with it, it leads to diffuse edema leading to cerebral ischemia [7]. In our case, the child presented consciousness disorders without neurological deficit. Usually the treatment of subdural hematoma in infants consists of a craniectomy with evacuation. This therapeutic strategy involves risks such as intraoperative hypovolemic shock and hematoma among others [8]. In our patient we noted after the evacuation of the subdural hematoma, postoperative complications such as intraparenchymal hematoma with fairly significant cerebral edema and mass effect on neighboring structures. We thus proceeded with success, with a decompressive craniectomy in order to prevent the installation of a cerebral ischemia, which would have been catastrophic. This article confirms that the CSF ventricular puncture, although being a simple procedure, is not without complications and should be strictly reserved for neurosurgeons only. In order to prevent accidents of hyper evacuation of CSF causing rupture of bridging veins by collapse of the cerebral parenchyma and the formation of a subdural hematoma.

## 4 Conclusion

Subdural hematoma in infants is often traumatic in origin. The rupture of the bridge veins is an uncommon cause of these hematomas. In the reported case, it follows a ventricular puncture of CSF. This simple gesture is not trivial and devoid of complications and should be performed by neurosurgeons only.

## Sources of funding

No funding was obtained for this study.

## Ethical approval

The study is exempted from ethical approval.

## Consent

Written informed consent was obtained from the patient for publication of this case report and accompanying images. A copy of the written consent is available for review by the Editor-in Chief of this journal on request.

## Registration of research studies

Not applicable.

## Author guarantor of the submission

Dr. Yakhya CISSE.

## Provenance and peer review

Not commissioned, externally peer-reviewed.

## Registration of research studies

Not applicable.

## CRediT authorship contribution statement

All the authors contributed to this work. Pr Seydou boubakar BADIANE

**Study conception and design**: Dr El Hadji Cheikh Ndiaye SY, Dr Yakhya CISSE, Dr Jean Michel NZISABIRA,.

**Data acquisition**: Dr Yakhya CISSE, Dr Pape Sandene NDIAYE, Dr Jean Michel NZISABIRA, Dr Ansoumane DONZO

**Analysis and data interpretation:** Dr Yakhya CISSE. Dr Jean Michel NZISABIRA,

**Drafting of the manuscript**: Dr El Hadji Cheikh Ndiaye SY, Dr Jean Michel NZISABIRA, Dr Yakhya CISSE.

## Declaration of competing interest

The authors claims no conflicts of interests.
